# Peri-Pubertal Emergence of UNC-5 Homologue Expression by Dopamine Neurons in Rodents

**DOI:** 10.1371/journal.pone.0011463

**Published:** 2010-07-08

**Authors:** Colleen Manitt, Cassandre Labelle-Dumais, Conrad Eng, Alanna Grant, Andrea Mimee, Thomas Stroh, Cecilia Flores

**Affiliations:** 1 Department of Psychiatry, Douglas Mental Health University Institute, McGill University, Montreal, Canada; 2 Department of Neurology and Neurosurgery, Montreal Neurological Institute, McGill University, Montreal, Canada; The Mental Health Research Institute of Victoria, Australia

## Abstract

Puberty is a critical period in mesocorticolimbic dopamine (DA) system development, particularly for the medial prefrontal cortex (mPFC) projection which achieves maturity in early adulthood. The guidance cue netrin-1 organizes neuronal networks by attracting or repelling cellular processes through DCC (deleted in colorectal cancer) and UNC-5 homologue (UNC5H) receptors, respectively. We have shown that variations in netrin-1 receptor levels lead to selective reorganization of mPFC DA circuitry, and changes in DA-related behaviors, in transgenic mice and in rats. Significantly, these effects are only observed after puberty, suggesting that netrin-1 mediated effects on DA systems vary across development. Here we report on the normal expression of DCC and UNC5H in the ventral tegmental area (VTA) by DA neurons from embryonic life to adulthood, in both mice and rats. We show a dramatic and enduring pubertal change in the ratio of DCC:UNC5H receptors, reflecting a shift toward predominant UNC5H function. This shift in DCC:UNC5H ratio coincides with the pubertal emergence of UNC5H expression by VTA DA neurons. Although the distribution of DCC and UNC5H by VTA DA neurons changes during puberty, the pattern of netrin-1 immunoreactivity in these cells does not. Together, our findings suggest that DCC:UNC5H ratios in DA neurons at critical periods may have important consequences for the organization and function of mesocorticolimbic DA systems.

## Introduction

The elaboration and establishment of organized patterns of neural connectivity during brain development rely on the concerted action of various permissive and instructive cues. Netrin-1, a member of the netrin family of guidance cues, participates in the organization of neural networks by attracting or repelling extending processes [1] [2] [3]. Two distinct families of netrin-1 receptors, the DCC (deleted in colorectal cancer) and UNC-5 homologue families (UNC5H; A-D), account for the bifunctional nature of netrin-1. Whereas DCC receptors mediate attraction, DCC-UNC5H receptor complexes, or UNC5H alone, mediate repulsion [1] [2] [4] [5] [6]. Consistent with their function, these netrin-1 receptors recruit downstream proteins that regulate cytoskeletal reorganization [7]. Recently, DSCAM (Down's syndrome cell adhesion molecule) has been identified as a novel netrin-1 receptor involved in signalling axon guidance [8] [9] [10]. However, netrin-1 mediated signal transduction via DSCAM is poorly understood.

High levels of DCC have been observed in cell body and terminal regions of developing and adult dopamine (DA) neurons [11] [12] [13] [14] [15] [16]. In addition, we and others have shown that DCC is expressed specifically by DA neurons in these regions in the adult rat and mouse brain [16] [17] [18], suggesting a role for DCC in the organization and function of DA systems. This hypothesis was confirmed by a series of studies we conducted recently in *dcc* heterozygous (+/−) mice showing that mice expressing reduced levels of DCC exhibit profound changes in mesocorticolimbic DA organization and function in adulthood. Indeed, adult *dcc* +/− mice exhibit sizeable increases in basal and amphetamine-induced DA activity in the medial prefrontal cortex (mPFC) and decreased amphetamine-induced DA release in the nucleus accumbens (NAcc) [17] [19]. Concomitantly, adult *dcc* +/− mice display altered mesocorticolimbic DA-mediated behaviors including blunted amphetamine-induced locomotion and reward, and resistance to amphetamine-induced deficits in prepulse inhibition [17] [19] [20]. Significantly, the *dcc* +/− dopamine and behavioral phenotypes are not evident before puberty [20], demonstrating a critical temporal window of vulnerability within the mesocorticolimbic DA system to the effects of reduced DCC levels.

Together, these findings indicate that netrin-1 receptors participate in the development of the mesocorticolimbic DA system and that their organizational effects occur at specific critical periods. Here we report on the normal profile of DCC and UNC5H expression by mesocorticolimbic DA neurons from embryonic life to adulthood in Sprague-Dawley rats and C57BL/6 (BL6) mice. First, using Western blot analysis, we demonstrate that the ratio of DCC to UNC5H expression switches toward UNC5H predominance during peri-pubertal age in DA somatodendritic regions. To capture possible changes in DCC and UNC5H expression by mesocorticolimbic DA neurons specifically, we conducted dual-immunofluorescence experiments at specific developmental stages. These stages were selected based on the selective changes that take place during the elaboration of the mesocorticolimbic DA circuitry [21] [22] [23] [24] [25]. We find that whereas DA neurons express DCC from embryonic life to adulthood, UNC5H expression by these cells emerges at puberty.

## Materials and Methods

### Animals

All experiments were preformed in accordance with the guidelines of the Canadian Council of Animal Care and all animal procedures were approved by the McGill University/Douglas Hospital Animal Care Committee (protocol number: 5084). All animals were kept on a 12 h light-dark cycle with ad libitum access to food and water.

#### Mice

C57BL/6J (BL6) mice were obtained from The Jackson Laboratory and bred in our animal colony. Mice were used at embryonic day (E) 13, 15, and 17; at birth (postnatal day (PND) 0), at post-weaning PND21±1; at peri-pubertal period PND33±2, and at adulthood PND75±15. For staging of embryos, the day of fertilization, assessed by the presence of a vaginal plug, was considered to be E0. For post-weaning to adulthood experiments, only male mice were used. ***Dcc −/− mice:*** For the experiments aimed at testing DCC antibody specificity (see below), *dcc* −/− embryos were generated by mating adult *dcc* +/− mice. Adult *dcc* heterozygous (+/−) mice, originally obtained from Dr. S. Ackerman (The Jackson Laboratory), were maintained on a BL6 background and bred in our animal colony. Targeted inactivation of the *dcc* gene was performed by disrupting exon 3, which encodes most of the protein's second immunoglobulin-like domain, by insertion of a neomycin resistance cassette using homologous recombination [26]. Southern and Western blot analyses were used to confirm proper targeting and complete loss of DCC protein, respectively [26]. ***Unc5c −/− mice***: For the experiments aimed at testing the specificity of the anti-UNC5C rabbit antiserum, brains dissected from adult *unc5c* −/− and +/+ mice were used (BL6; Cg-Unc5c^rcmTg(Ucp)1,23KZ/Slac^; obtained from Dr. S. Ackerman, The Jackson Laboratory). The mutant allele of the *unc5c* locus was generated by a transgene insertion that results in complete loss of *unc5c* transcript [27]. These mice were from a hybrid BL6/SJL background because −/− progeny from a pure BL6 background do not typically survive to adulthood.

#### Genotyping

The targeted and wild-type *dcc* alleles were amplified using an annealing temperature of 54°C for 30 cycles with the following oligonucleotides: DCC code: GGT CAT TGA GGT TCC TTT, DCC rev: AAG ACG ACC ACA CGC GAC, and DCC Neo: TCC TCG TGC TTT ACG GTA TC. The targeted and wild-type *unc5c* alleles were amplified using an annealing temperature of 54°C for 40 cycles using the following oligonucleotides: Tipsy head CAG GAG AAG ATA CAT TTA ACC AC, Tipsy tail GAC AGA AGA GCA TAG CAT TCAC, Asp-2M13RA CAC TCT ATG GAA ATG GCT GAA T, Asp-2M13RB GTC CTC CAA TCC AAG AAC TG.

#### Rats

The development of midbrain DA neurons in mice precedes that of rats by two days (Bayer et al., 1995). Thus, we collected embryos at E15, 17, and 19 and at birth (PND0) from timed pregnant Sprague-Dawley rats obtained from Charles River. Post-weaning (PND21), peri-pubertal (PND33) and adult (PND90) Sprague-Dawley rats were also obtained from Charles River.

### Rationale for choosing specific developmental ages

The embryonic and postnatal developmental stages used for the characterization of the expression pattern of DCC and UNC5H by rodent midbrain DA neurons were selected on the basis of the development of the DA system in the rat [21] [22] [24]. Briefly, in the rat, the first DA neurons are generated in the ventral midbrain between E12 and E15 [22] [28]. Shortly after their specification, midbrain DA neurons start sending projections toward their targets. At E13/E14 these neurons extend axons rostrally and, by E15, they start innervating the ganglionic eminence (i.e. developing striatum). At E16/E17 some DA fibers grow beyond the ganglionic eminence to reach the cortical subplate. By E19/E20, DA fibers innervate the cortical plate of the developing medial prefrontal cortex. At birth, the number of DA fibers in deep layers of the cortex increase dramatically. Mesolimbic DA innervation reaches adulthood levels three weeks after birth. In contrast, maturation of mesocortical DA innervation proceeds until early adulthood and exhibits significant remodeling during the post-weaning (PND23) and peri-pubertal period (PND35) [21] [22].

### Primary antibody characterization

Please refer to Table 1 for a list of all primary antibodies used for immunofluorescence staining. The monoclonal tyrosine hydroxylase (TH) antibody (Chemicon International, Temecula, CA; cat # MAB318) was raised in mouse and recognizes specifically an epitope on the regulatory N-terminus of TH [29]. On Western blot, it recognizes a single band of ∼60 kDa, which corresponds to the predicted molecular weight for TH and does not react with other closely related catecholamine enzymes including dopamine-β-hydroxylase, phenylalanine hydroxylase, tryptophan hydroxylase, dehydropteridine reductase, sepiapterin reductase and phenethanolamine-M-methyl-transferase (manufacturer's technical information). This antibody was shown to result in a staining pattern of rat striatal dopaminergic terminals similar to that described previously [30] and to stain mesencephalic DA neurons in the adult rat [31]. The rabbit polyclonal anti-TH antibody (Chemicon International; cat # AB152) was raised in rabbit against denatured TH from rat pheochromocytoma. On Western blot, this antibody recognizes a single band of approximately 60 kDa molecular weight corresponding to the TH protein (manufacturer's technical information). This antibody labels midbrain DA neuron cell bodies and terminals in wild-type mice, but not in mice that have a non-functional *Th* gene [32]. The chicken polyclonal anti-TH antibody (Abcam, Cambridge, MA; cat # AB53417) was raised in chicken against two synthetic peptides corresponding to different regions of TH with sequences shared between the mouse (P24529) and human (P01701). This antibody recognizes a specific band of approximately 59 kDa on Western blot (manufacturer's technical information) and results in a staining pattern of rat midbrain DA neurons identical to that observed with the two other TH antibodies used in this study. The chicken anti-mouse netrin-1 antibody (1∶7000, Novus Biologicals, Littleton, CO) was tested by Western blot. Lysates from whole rat brain were run alongside rat liver, which does not express netrin-1 [33]. A band of ∼75 kDa was detected in adult rat brain, consistent with the predicted molecular weight of rat netrin-1. This band was not detected in liver (data not shown). The rabbit anti-DCC polyclonal antiserum (2744, [34] a gift from Dr. H.M. Cooper) was raised against the C-terminal peptide (SEESHKPTEDPASV) corresponding to amino acids 1406–1419 of the mouse DCC protein [35]. The specificity of the antibody has been demonstrated previously by preincubating the antiserum with the peptide antigen prior to incubating sections [36].

**Table 1:** List of primary antibodies used for immunofluorescence staining.

Antigen	Immunogen	Manufacturer	Dilution used
Tyrosine Hydroxylase	Tyrosine hydroxylase purified from PC12 cells	Chemicon, Temecula, CA; mouse monoclonal, MAB318	1∶300
Tyrosine Hydroxylase	SDS-denatured tyrosine hydroxylase from rat pheochromocytoma	Chemicon, Temecula, CA; rabbit polyclonal, AB152	1∶500
UNC5H (UNC-5 homologues)	Synthetic peptide, aa 605-877 at C terminus of UNC5H3 (RCM), a region sharing high homology in aa identity between mammalian UNC-5 homologues.	Dr. Tony Pawson, University of Toronto, rabbit polyclonal, 6th bleed of rabbit #52	1∶5000 for free floating sections 1∶7500 for sections apposed to slide
DCC (deleted in colorectal cancer)	Truncated recombinant human DCC protein containing the intracellular domain, clone G97-449	BD Pharmingen, Missisauga, ON; mouse monoclonal, cat# 554223	1∶500 for free floating sections 1∶400 for sections apposed to slides
Tyrosine hydroxylase	Synthetic peptides conjugated corresponding to different regions of the TH gene product shared between the mouse (P24529)and human (P07101).	Abcam, Cambridge, MA; chicken polyclonal, cat # AB53417	1∶1000
Netrin-1	synthetic peptide corresponding to mouse and human netrin-1 gene product,	Novus Biologicals, Littleton, CO cat# NB 100-1605	1∶3500 for free floating sections
DCC (deleted in colorectal cancer)	C-terminal peptide (SEESHKPTEDPASV) corresponding to amino acids 1406–1419 of the mouse DCC protein	Dr. Helen M. Cooper, University of Queensland (#2744)	1∶2000 for free floating sections

The monoclonal DCC antibody (BD Pharmingen, Missisauga, ON; cat# 554223, clone G97-449) was raised in the mouse against a truncated recombinant protein containing the intracellular domain of the recombinant human DCC (C terminal 338 amino acids, aa 1110-1448). This antibody recognizes a single band of ∼185 kDa on Western blot, consistent with the molecular weight of DCC (manufacturer's technical information). We determined DCC antibody specificity by performing Western blot analysis on E17 whole brain tissues lysate from *dcc* −/− and +/+ mice and by immunohistochemistry on midbrain sections of E17 *dcc* −/− embryos.

The polyclonal UNC5H antiserum, kindly provided by Dr. Pawson (University of Toronto), was raised against a GST fusion protein expressing the region between the ZU-5 and the death domain of UNC5C (RCM) (residue 605–877, GST-RCMZO-DD, 6th bleed of rabbit #52). The 272-amino-acid peptide antigen used to generate this antiserum shares high amino acid identity with the corresponding sequence in other mammalian UNC-5 homologues. UNC5H antibody specificity has been demonstrated by immunoprecipitation, Western blotting and immunostaining of HEK-293 cells transfected with *unc5c* cDNA [37]. Furthermore, the antiserum has been shown to recognize recombinant UNC5A, UNC5B or UNC5C by Western blot, and was shown to detect a single band of 135 kDa on Western blots of rat spinal cord homogenates [38]. Here we further characterized this antibody by assessing whether the antiserum could also recognize other endogenous UNC-5 homologues from brain tissue lysates, in addition to UNC5C. Western blot analysis was performed on tissue lysates of cerebella dissected from *unc5c* −/− and +/+ adult mice. Immunohistochemistry on tissue sections through the ventral tegmental are (VTA) of adult *unc5c* −/− and +/+ mice was also performed as described below.

### Characterization of UNC-5 homologue mRNA expression by RT-PCR

PND60 Sprague-Dawley rats were killed by decapitation following carbon dioxide inhalation, and brains were dissected and rapidly frozen in 2-methylbutane chilled with dry ice. Bilateral punches (0.5 mm in diameter) of the ventral tegmental area (VTA) were excised from coronal sections spanning Plates 41 to 43 of Paxinos and Watson (1998) (500 µm thickness). Total RNA was isolated from tissue punches by trizol extraction (Invitrogen, San Diego, CA). First strand cDNA was synthesized by using M-MLV reverse transcriptase and oligo(dt)15 primers, and the cDNA was amplified by PCR using the primer pairs listed below. Primers were annealed at 68°C for 35 cycles. The size and corresponding coding sequence of the predicted amplification products are as follows: rat *unc5a*: a portion of the intracellular domain, transmembrane domain, and a portion of the extracellular domain (575–1481; 907 bp), forward primer: 5_ GGA ATT CCC TCC CTC GAT CCC AAT GTG T 3_, reverse primer: 5_ TCC CCG CGG GGC AGG GAA CGA AAG TAG T 3_; rat *unc5b* (339–1106;768 bp), forward primer: 5_ GCT CTA GAG TCG CGG CAG CAG GTG GAG GAA 3_, reverse primer: 5_ GGA ATT CAG GGG GCG GCT TTT AGG GTC GTT 3_. Rat *unc5c* (1096−1761; 666 bp), forward primer: 5_GAT GGC AGG TGG ACT TCG TG3_, reverse primer: 5_GTT GAA GGT GCC AAA CGC TG3_. rat unc5d: (1691–2577; 887 bp), forward primer: 5_ GAA GAA GAG AAC GCA GCA G 3_, reverse primer: 5_ TCG TGT GTG TCC TCC CAA TC 3_. RT-PCR amplification products were separated by agarose gel electrophoresis.

#### Tissue preparation and Western blot analysis

Whole brains from PND23, PND35 and PND90 male rats were flash frozen in 2-methylbutane. Bilateral punches of the ventral tegmental area (VTA) were excised from 1mm thick coronal sections of PND23, PND35 and PND90 rat brains as previously described [17] [18] [19] [18], [ 20], [ 39]. Midbrains from E17 and PND0 rats were dissected and flash frozen in 2-methylbutane. The level of expression of DCC, UNC5H in the VTA of PND23, PND35 and PND90 rats and in the midbrain of E17 and PND0 rats was evaluated using Western blot analysis as described previously [17] [18]. Briefly, protein samples from VTA and midbrain homogenates (25 µg) were resolved on a 7.5% SDS-PAGE and transferred to nitrocellulose membranes (Amersham Biosciences, Freiburg, Germany). Membranes were incubated with primary antibodies against DCC (1∶1000), UNC5H (1∶7500), and tubulin (1∶4000, mouse monoclonal, Sigma). Expression of all proteins was assessed using the same membrane, and tubulin was used as a loading control. Importantly, the primary antibodies against DCC and UNC5H used in this procedure were the same as the ones described in the immunohistochemistry section. Immunoreactivity was visualized using peroxidase-conjugated secondary antibodies (Vector Laboratories) and chemiluminescence (Perkin Elmer, Waltham, MA, USA).

### Immunohistochemistry

To collect embryonic brains, pregnant females were killed by decapitation, embryos were surgically removed from the uterus, decapitated and their brains dissected in PBS and fixed by immersion in 4%PFA and 0.2% picric acid in 0.1 M phosphate buffer, overnight at 4°C. Fixed embryonic brains were then cryoprotected overnight at 4°C in 30% sucrose in PBS. Cryoprotected embryonic brains were embedded in Tissue-Tek O.C.T. compound (Sakura) and frozen in 2-methylbutane chilled with dry ice. PND0 brains were processed as described for embryonic brains. 20 µm coronal sections of embryonic and PND0 brains were cut using a cryostat. Sections were mounted directly on superfrost slides (Fisher Scientific), air-dried and stored at −80°C until use. Juvenile, post-weaning and adult animals were anesthetized with an overdose of sodium pentobarbital (>75 mg/kg i.p.) and were perfused intracardially with 0.9% saline followed by a fixative solution (4% paraformaldehyde (PFA) and 0.2% picric acid in 0.1 M phosphate buffer). Brains were dissected from the skull, post-fixed in the same fixative for 45 minutes at 4°C and cryoprotected in sucrose (30% sucrose in phosphate buffered saline (PBS)) overnight at 4°C. The following morning, tissue was rapidly frozen by immersion in 2-methylbutane (Fisher Scientific, Hampton, NH) chilled with dry ice. Frozen brains were immediately sectioned at 40 µm using a Leica SM2000-R sliding microtome and collected in PBS. Free-floating sections were used immediately for immunohistochemical processing.

Tissue sections were processed for immunofluorescence as described previously [17] [18]. Briefly, rat brain sections were collected and rinsed in PBS and incubated in blocking solution (rat: 2% bovine serum albumin, 0.2% Tween-20 in PBS; mouse; M.O.M. kit, Vector Laboratories, Burlingame, CA, USA) for 2 hours at room temperature (RT). Sections were incubated overnight at 4°C with combinations of primary antibodies diluted in blocking solution. The combinations of following primary antibodies were used: mouse monoclonal anti-DCC (1∶500, Pharmigen, Mississsauga, Ontario, Canada, Cat# 554223), rabbit polyclonal anti-TH (1∶500, Chemicon, Temecula, CA, USA, Cat# AB152), rabbit UNC5H antiserum (1∶5000, provided by Dr. Tony Pawson, University of Toronto), and mouse monoclonal anti-TH (1∶300, Chemicon, Cat# MAB318), polyclonal chicken anti-mouse netrin-1 (1∶3500, Novus Biologicals, Littleton, CO), or polyclonal rabbit anti-DCC (2744; 1∶2000, a gift from Dr. H.M. Cooper, University of Queensland). Immunostaining was visualized with either Alexa Fluor 350, Alexa Fluor 488 or Alexa Fluor 555-conjugated secondary antibodies raised in goat (1∶500, Molecular Probes, Eugene, OR, USA).

As a negative control, adjacent sections were processed as described above except that they were incubated overnight at 4°C in blocking solution without primary antibodies. To test for secondary antibody specificity, sections were incubated with either only DCC, UNC5H, or netrin-1 primary antibody and, subsequently, with combinations of anti-rabbit, anti-mouse and anti-chicken Alexa Fluor-conjugated antibodies. Immunoreactivity was only observed for the secondary antibody raised against the species in which the primary antibody was generated.

### Microscopy and image analysis

Immunofluorescence was visualized using either a 1) Leica DM4000B microscope equipped with a Ludl XYZ motorized stage and filter cubes appropriate for detection of Alexa Fluor 350, 488 and 555. Images were captured using a digital Microfire camera and PictureFrame software (Microbrightfield, VT, USA) or 2) a Nikon PCM2000 laser-scanning confocal microscope equipped with argon (488 nm excitation; 10% neutral density filter) and HeNe (543 nm excitation) lasers. Confocal images of Alexa Fluor 488 and 555 were obtained simultaneously, below saturation levels, with minimal gain and contrast enhancement.

The presence of immunostained cells in the VTA from post-weaning to adulthood was examined on coronal sections corresponding to sections spanning Plates 55 to 63 of the mouse brain atlas [40] and sections spanning Plates 40 to 46 of the rat brain atlas [41]. For embryonic ages and for PND0, coronal sections containing midbrain DA neurons were examined according to the atlas of the developing mouse [23], and rat [42] brain.

For all fluorescence pictures, adjustment for brightness and contrast in addition to adjustment of tonal range for each individual RGB channel were performed using Adobe Photoshop Creative Suite edition (Adobe System, San Jose, CA).

## Results

### Peri-pubertal switch in the relative ratio of DCC to UNC5H netrin-1 receptor expression in somatodendritic DA regions

**Figure 1:**
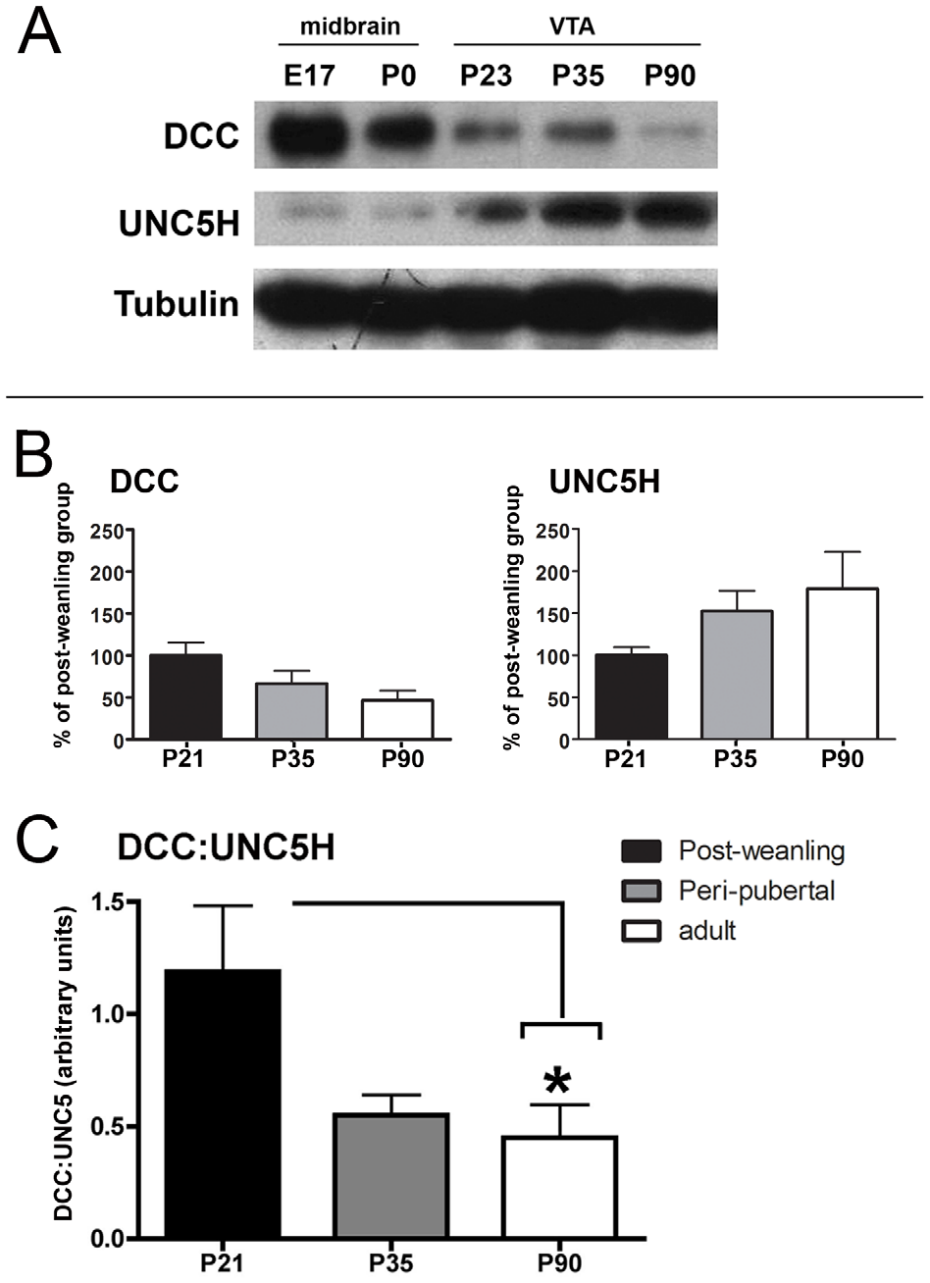
Developmental shift in the expression pattern of netrin-1 receptors in the VTA. A) Western blot showing netrin-1 receptor expression in tissue lysates from rat midbrains at E17 and PND0, and in lysates from PND23, PND35, and PND90 rat VTA. Note that the expression patterns of DCC and UNC5H change in opposite directions throughout development. Whereas DCC expression decreases with age, UNC5H expression increases. B–D) Quantitative analysis of western blots performed on lysates from VTA of PND21, PND35, and PND90 mice. B) DCC levels progressively decreased from post-weaning to adulthood. Conversely, UNC5H levels progressively increased between juvenile and adult periods. One-way ANOVAS revealed no statistical significance (DCC: F *_(2, 14)_* = 3.50, *p* = 0.06; UNC5H: *F*_(2, 20)_ = 2.54, *p* = 0.10). C) Analysis of DCC and UNC5H expression levels when expressed as a DCC:UNC5H ratio within each sample. A dramatic shift toward UNC5H predominance occurs between puberty and adulthood. (Fig. 1C; one-way ANOVA: *F _(2,13)_*  =  4.42; *p*  =  0.03; post hoc Tukey's HSD tests revealed a critical q = 3.73 (α =  0.05). The comparison between PND21 and PND35 yielded a q(13)  =  3.47, *p*>0.05. The comparison between PND21 and PND90 yielded a q(13)  =  3.84, *p*<0.05. The comparison between PND35 and PND90 yielded a q(13)  =  0.54, *p*>0.05). Animals studied in experiment A: n  =  3 per group (replicated 3 times); animals studied in experiments B–D: post-weaning n =  9, peri-pubertal n  =  8, adult n = 6.

Figure 1A shows the expression of DCC and UNC5H in the VTA from embryogenesis to adulthood. At all ages, a single ∼185 kDa band was observed with the DCC antibody and a single ∼135 kDa band was detected with the UNC5H antiserum. Whereas DCC immunoreactivity was high in the rodent midbrain during embryonic development, UNC5H immunoreactivity was barely detectable. At birth, DCC levels continued to be high compared to UNC5H expression, but were reduced in comparison to embryonic DCC levels. At post-weaning, a substantial reduction in DCC expression was observed as well as a noticeable increase in UNC5H immunoreactivity. From peri-pubertal period to adulthood, DCC levels continued to decrease dramatically and UNC5H was expressed at substantially higher levels than DCC.

Our previous work implicates netrin function in the development of the mesocorticolimbic system at puberty [17] [19] [20]. A quantitative analysis was performed on data obtained from Western blots of VTA punches at post-weaning (PND 21), puberty (PND 35), and adulthood (PND 90) (Fig. 1B, C). Whereas expression of DCC from PND21 to adulthood appeared to decrease, the opposite seemed to occur with UNC5H expression. Because the immunoblots were developed separately, we first analyzed these data using separate one-way ANOVAs for each receptor (Figure 1B; DCC: F *_(2, 14)_* = 3.50, *p* = 0.06; UNC5H: *F*_(2, 20)_ = 2.54, *p* = 0.10). However, because the ratio of DCC:UNC5H netrin receptor expression is important in determining the response to netrin-1 [5] [43] [44] [45] [46] [47] [48], we examined whether a sift in the ratio occurs around puberty in the VTA. When the relative levels of receptor expression within each sample were expressed as a DCC:UNC5H ratio, a significant and dramatic shift toward UNC5H predominance occurred from PND21 to adulthood (Fig. 1C; one-way ANOVA: *F_(2,13)_* = 4.42; *p* = 0.03; post hoc Tukey's HSD tests revealed a critical q = 3.73, α = 0.05. The comparison between PND21 and PND35 yielded a q(13) = 3.47, *p*>0.05. The comparison between PND21 and PND90 yielded a q(13) = 3.84, *p*<0.05. The comparison between PND35 and PND90 yielded a q(13) = 0.54, *p*>0.05). Together, these results indicate that the peri-pubertal period marks a transition toward a relative DCC:UNC5H ratio of netrin-1 receptor expression that is maintained into adulthood.

### DCC and UNC5H Antibody specificity

Western blot analysis indicated that netrin-1 receptor expression in the VTA is developmentally regulated, and that a switch in the relative ratio of DCC:UNC5H expression in the VTA toward UNC5H predominance occurs around puberty. We next determined the normal pattern of expression of DCC and UNC5H in VTA DA neurons from embryogenesis to adulthood in both mice and rats.

The specificity of the DCC antibody was confirmed by Western blot performed on whole brain homogenates of E17 *dcc* −/− mice and +/+ controls (Fig. S1A). As we previously showed in rat and mouse brains [19] [17] [18], the DCC antibody detected a single ∼185 kDa band in brains of E17 +/+ mice. However, no immunoreactive band was detected in brains of *dcc* −/− embryos. DCC immunofluorescence on midbrain sections of E17 *dcc* −/− embryos revealed no immunoreactive signal (Fig. S1B), whereas high levels of DCC have previously been reported in wild type mice [11] [12].

It is important to mention that similar to what we observed in +/+ mouse embryos, TH-immunopositive neurons were also present in the ventral midbrain region of *dcc* −/− embryos (Fig. S1B), indicating that DCC-mediated netrin-1 signalling is not required for the formation and differentiation of midbrain DA neurons.

The specificity of the UNC5H antiserum has been demonstrated previously [37] 38. This antiserum was raised against a peptide sequence in the mouse UNC5C cytoplasmic domain, and its specificity for UNC5C was confirmed by immunoprecipitation, Western blot analysis, and immunostaining of HEK-293 cells expressing *unc5c* cDNA [37]. This antiserum was also shown to bind recombinant UNC5A and UNC5B by Western blot analysis [38].

We now further characterized the specificity of this antiserum by Western blot using whole cerebella from adult *unc5c* −/− and +/+ mice ([27], Fig. S1C). Adult mouse cerebellum expresses all the identified mammalian UNC-5 homologues (*unc5a* & *unc5b*, Leonardo et al., 1997; *unc5c*, Ackerman et al., 1997; *unc5d*, unpublished observations, RT-PCR). The antiserum detected a ∼135 kDa band in both *unc5c* −/− and +/+ mice, indicating that it recognizes other endogenous UNC-5 homologues, in addition to UNC5C. Immunohistochemistry conducted in cerebellar tissue from *unc5c −/−* and +/+ mice revealed no differences in the labelling pattern (Fig. S1D).

We also performed RT-PCR experiments on discrete tissue punches taken from the VTA of adult wild-type mice (Fig. S1E). *unc5c* and *unc5d* were found to be expressed in the VTA. Because of this finding, we examined the pattern of immunofluorescence produced by the antiserum in the VTA of adult *unc5c* −/− and +/+ mice. However, again there was no difference in the pattern of immunoreactivity between genotypes, confirming that the antiserum recognizes the two UNC-5 homologues expressed in the adult VTA, namely UNC5C and UNC5D (Fig. S1F). Furthermore, the similar pattern of UNC5H immunoreactivity between the two genotypes suggests that UNC5C and UNC5D are co-expressed. The potential functional significance of UNC5D, or UNC5C and UNC5D co-expression, by DA neurons is presently unknown.

### During embryonic development, DA neurons express DCC, but not UNC5H

**Figure 2:**
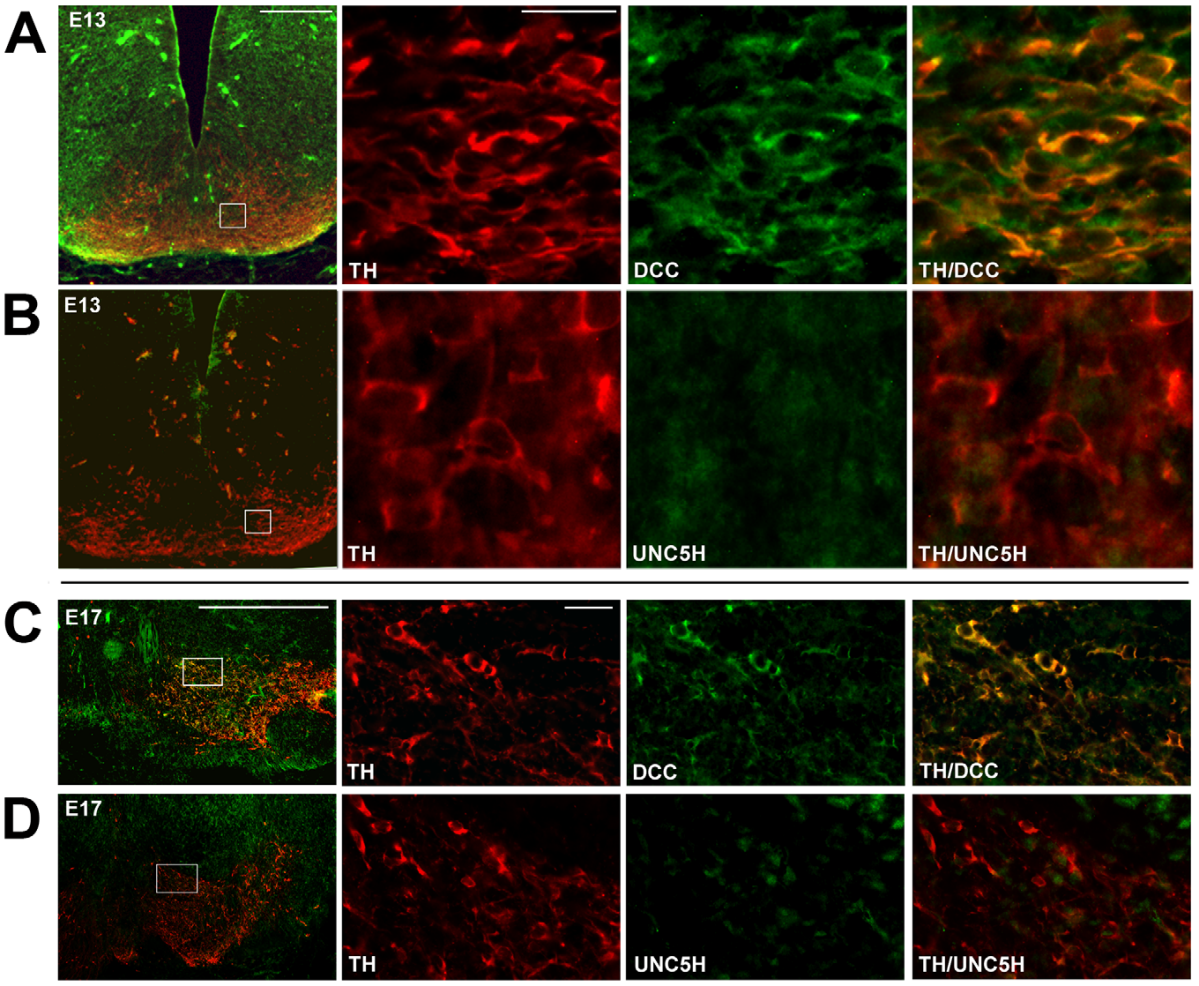
Netrin-1 receptor expression in embryonic VTA dopamine neurons. Low magnification micrographs in the first panel of A through D illustrate coronal ventral midbrain sections from E13 wild-type (A,B) and E17 wild-type (C,D) mouse embryos immunolabelled with DCC (A,C; green) or UNC5H (B,D; green) and TH (red). Regions surrounded by a box represent the location within the embryonic VTA (The Altlas of the Developing brain, [23]) that is presented in neighboring higher magnification panels. A) Widespread expression of DCC is detected in the embryonic midbrain, with DCC and TH co-localizing in the ventral midbrain. B) Only scarce expression of UNC5H was observed in the embryonic ventral midbrain and no co-localization of UNC5H and TH was detected. In all pictures, the dorsal aspect of coronal sections is on top. Scale bars: 250 µm (low magnification panels) and 25 µm (high magnification panels). C–D) Netrin-1 receptor expression in E17 mouse midbrain dopamine neurons. Digitized images of coronal ventral midbrain hemisections from E17 wild-type mouse embryos indicate that DCC (C), but not UNC5H (D), is expressed in TH immunopositive neurons of the VTA in E17 mouse embryos. Dorsal is on top, lateral on the right, and medial is on the left. Similar results were obtained at E15 (data not shown). Animals studied in experiments A–D: n = 7 per group, Scale bars: 250 µm (low magnification panels) and 25 µm (high magnification panels).

Netrin-1 receptor expression was first assessed in the embryonic VTA at three different stages. Consistent with previous reports showing ubiquitous and specific expression of *dcc* mRNA [11] [12], DCC immunoreactivity was widespread in the embryonic mouse (Fig. 2) and rat (Fig. S2) VTA. Indeed, in both mouse (E13, E15, E17) and rat embryos (E15, E17, E19), DCC was highly expressed throughout the ventral midbrain region where the A9 (SN) and A10 (VTA) DA neurons are located (mouse: Fig. 2; rat: Fig. S2). In fact, most TH-positive neurons were found to express DCC at all embryonic stages examined and throughout the midbrain rostro-caudal axis (Fig. S2). These findings are consistent with previous reports suggesting expression of DCC by developing DA neurons in rats and mice [11] [12] [15]. In contrast, only scarce UNC5H expression was observed in the embryonic VTA (mouse: Fig. 2; rat: Fig. S2). No TH/UNC5H co-expression was observed in the ventral midbrain throughout the rostro-caudal axis at any of the embryonic stages analyzed (Fig. S2).

Qualitative analysis of the VTA throughout the rostro-caudal axis revealed high expression of DCC by TH-positive neurons at birth in both mice and rats. However, UNC5H immunofluorescence remained consistently low and no TH/UNC5H-positive cells were found in this region (Fig. S3).

### Netrin-1 receptor expression by DA neurons at post-weaning

**Figure 3:**
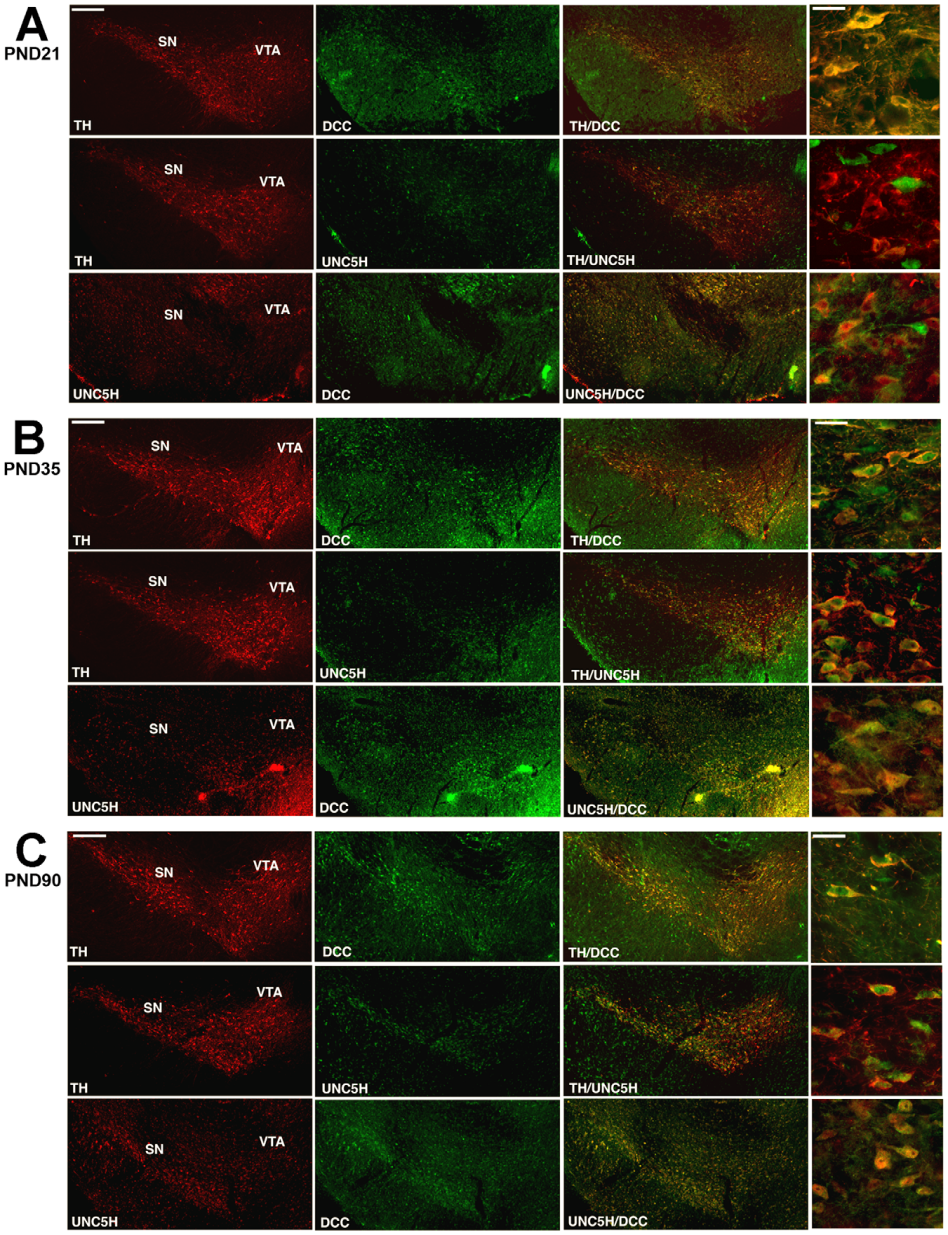
Netrin-1 receptor expression in post-weaning, peri-pubertal and adult mice. Digitized images of coronal midbrain hemisections from PND21 (A), PND33 (B) and adult (C) mice. In all pictures, dorsal is on top, lateral on the left, and medial on the right. DCC is expressed in many TH immunopositive neurons in VTA from post-weaning to adulthood. UNC5H expression is only present in some TH positive cells at post-weaning but is detected in many DA neurons from the peri-pubertal period to adulthood. DCC and UNC5H expression were co-localized in many VTA cells from post-weaning to adulthood. Panels on far right illustrate the immunohistochemical labeling patterns at higher magnification within the VTA. Animals studied in experiments A–C: n = 6 per group. Scale bars: 250 µm and 25 µm (high magnification).

DCC continued to be expressed by TH-immunoreactive neurons in the VTA during the post-weaning period in both mouse (Fig. 3A) and rat (rostro-caudal axis; Fig. S4). UNC5H immunofluoresence was observed to be increased in the VTA relative to the earlier developmental stages examined. Close inspection revealed emergence of sparse TH/UNC5H double-labelled cells. Most UNC5H positive cells also expressed DCC, although DCC and UNC5H were occasionally not co-expressed.

### Peri-pubertal switch in netrin-1 receptor expression by DA neurons

A dramatic emergence in TH/UNC5H double-labelled neurons was observed in the VTA of peri-pubertal mice (Fig. 3B) and rats (rostro-caudal field; Fig. S5). Consistent with this, expression of DCC and UNC5H by single VTA neurons also increased. These findings show that the peri-pubertal period is marked by an increase in UNC5H expression by DA neurons.

### DCC and UNC5H expression by DA neurons in adulthood

In agreement with our previous findings [17] [18], DA neurons in the VTA continue to express DCC and UNC5H in adulthood in both mice (Fig. 3C) and rats (Fig. S6). As reported previously [16], and similar to what we observed in peri-pubertal animals, TH/DCC and TH/UNC5H positive neurons were present in different subregions of the VTA; the parabrachial pigmented nucleus (PBP), interfascicular nucleus (IF), and paranigral nucleus (PN) (Paxinos and Watson, 1998). Not all TH positive neurons expressed UNC5H and this is true throughout the rostro-caulal extent of the VTA (Fig. S6).

**Figure 4:**
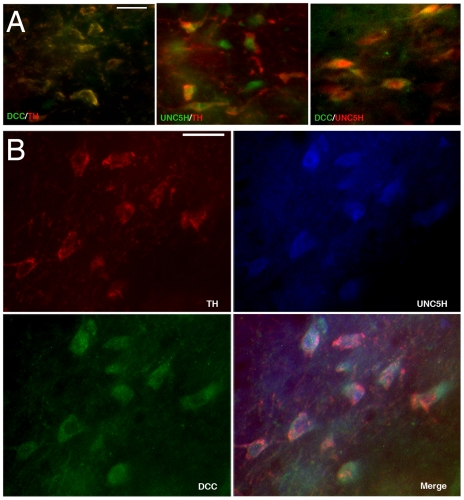
Netrin-1 receptor co-expression in adult midbrain DA neurons. Digitized images of the VTA from coronal midbrain hemisections from PND90 rats. In all pictures, dorsal is on top, lateral on the left, and medial on the right. A) High magnification images showing co-expression of DCC/TH, TH/UNC5H and DCC/UNC5H in VTA cells. Scale bar: 25 µm. B) Single VTA DA neurons express both DCC and UNC5H. High magnification images of VTA cells triple-labeled for TH, DCC, and UNC5H from PND90 rat coronal midbrain sections. Animals studied in experiments: A: n = 3, B: n = 2). Scale bar: 25 µm.

There was a high degree of DCC/UNC5H co-localization in the VTA, but some cells expressed only one type of receptor (Figs. 3C). To determine whether single VTA DA neurons indeed co-expressed UNC5H and DCC, we performed triple-labelling immunofluorescence in adult rat brain. As shown in Figure 4, single TH-positive neurons express both DCC and UNC5H.

### Netrin-1 is expressed by DA neurons before and after puberty

We have identified a pubertal shift in DCC and UNC5H expression by DA neurons. Lastly, we conducted experiments to determine whether netrin-1 is expressed in the somatodendritic DA region, and whether its distribution pattern also changes from post-weaning to adulthood.

**Figure 5:**
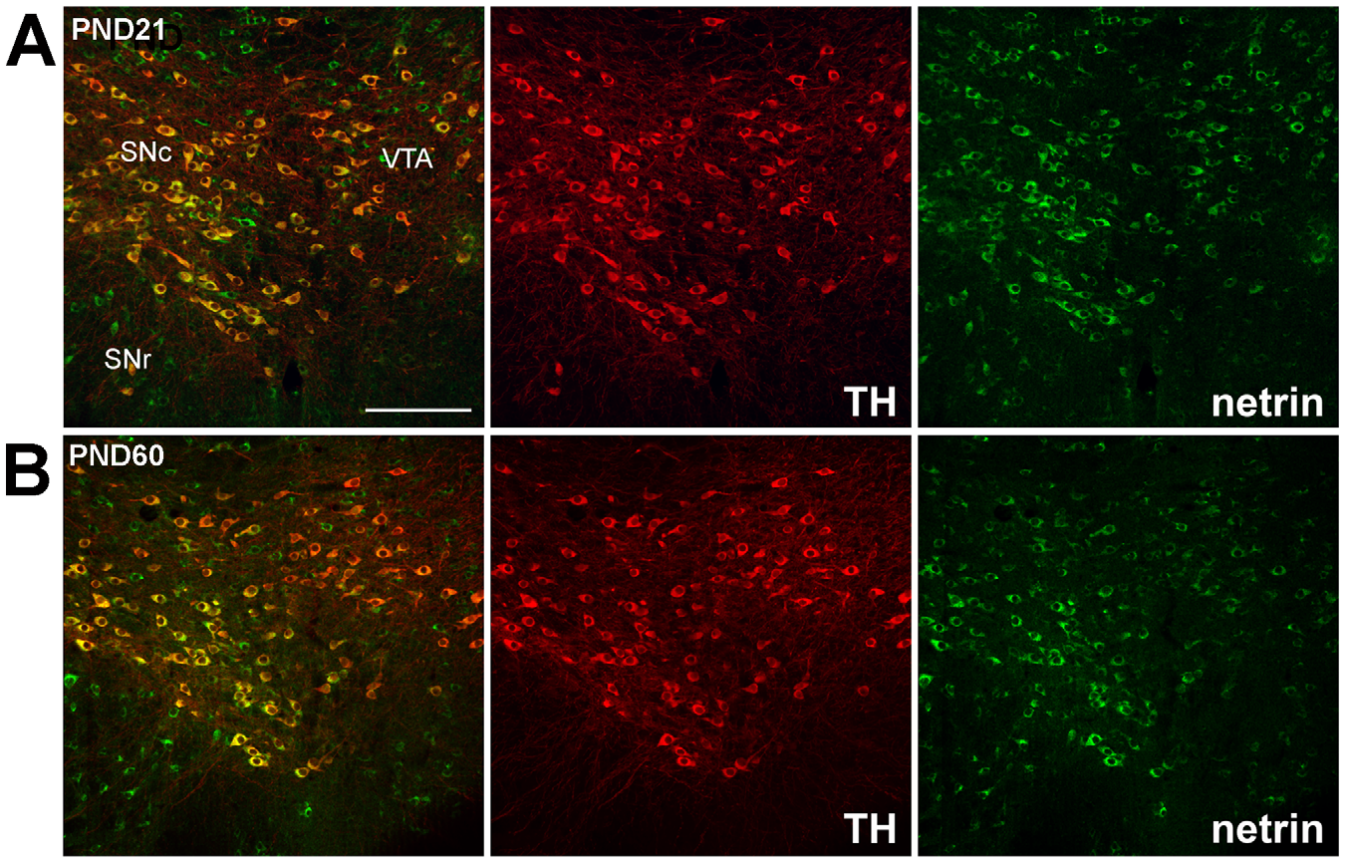
Netrin immunoreactivity in the midbrain before and after puberty. In all pictures, dorsal is on top, lateral on the left, and medial on the right. Micrographs of coronal midbrain hemisections from PND21 and PND60 mice immunolabeled with netrin-1 and TH immunoreactivity. The pattern of co-labeling is similar at both ages. In the VTA, a large proportion of TH-positive DA neurons are co-labeled with netrin-1 immunoreactivity. Several netrin+/TH- cells were also present in the VTA. Animals studied in experiments A and B: n = 5 per group. Scale bar: 250 µm.

**Figure 6:**
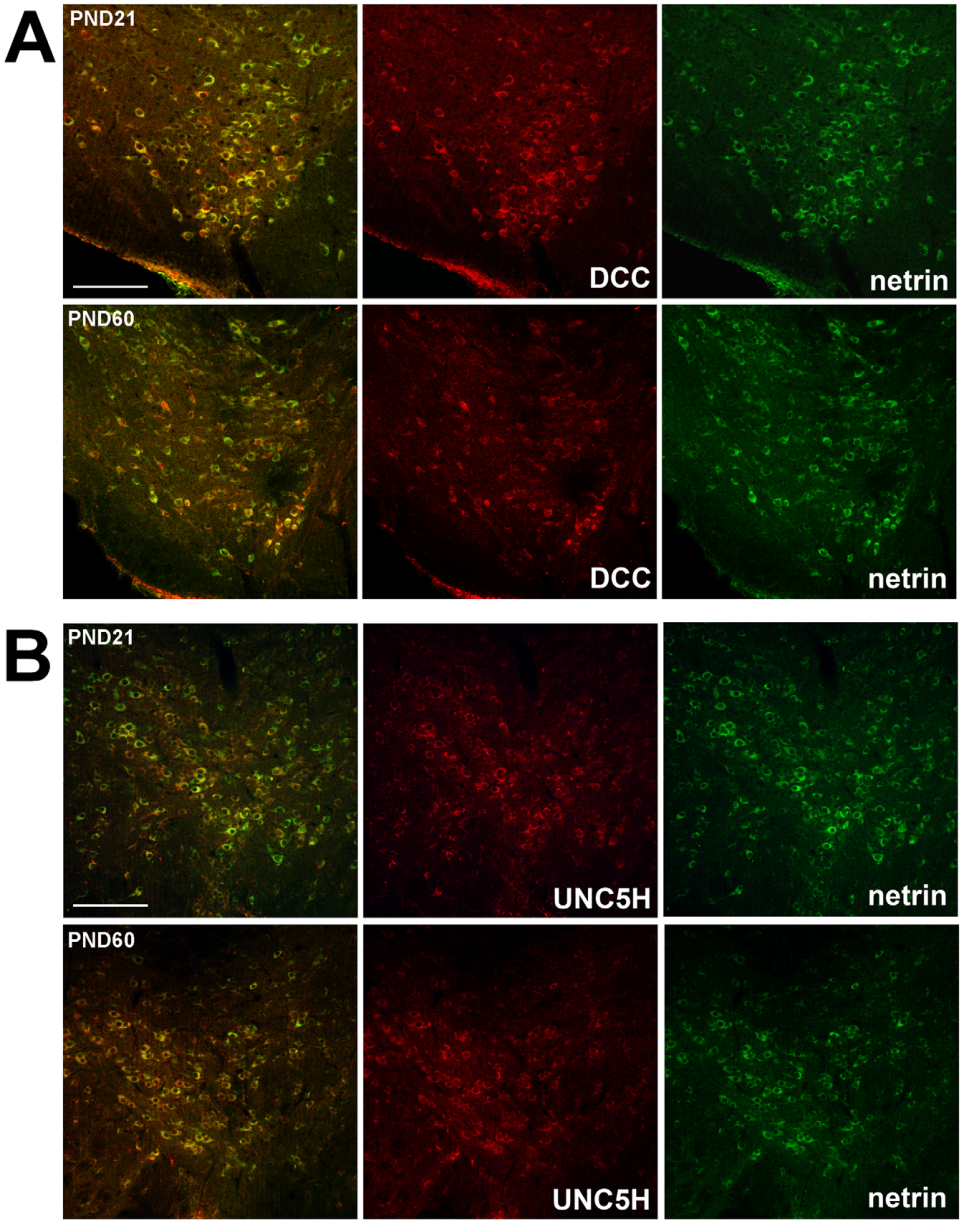
Netrin-1 and netrin-1 receptor co-labeling. Micrographs of coronal midbrain hemisections from PND21 and PND60 mice immunolabeled for netrin-1 and the netrin-1 receptors. Dorsal is on top, lateral on the left, and medial on the right. A) Micrographs of VTA cells co-labeled with netrin-1 and DCC immunoreactivity at PND21 and PND60. The number of netrin-1 positive cells that co-express DCC decreases after puberty. B) Micrographs of VTA cells co-labeled with netrin-1 and UNC5H immunoreactivity at PND21 and PND60. The number of netrin-1 positive cells that co-express UNC5H increases after puberty. Animals studied in experiment A: n = 7 per group. Scale bar: 250 µm.

Figure 5 illustrates the distribution of netrin-1 immunoreactivity in the VTA of juvenile and adult mice. Double-labelling with TH indicated that many DA neurons are netrin-1 immunoreactive at both ages (Fig. 5). Netrin-1 positive/TH immunonegative cells were also observed in the VTA at both ages (Fig. 5). Both DCC and UNC5H positive cells co-labelled with netrin-1 (Fig. 6). Fewer netrin-1/DCC positive cells were detected in adulthood relative to post-weaning. In contrast, the number of netrin-1 positive cells that co-labelled with UNC5H appeared to increase from post-weaning to adulthood. Although netrin-1 is a secreted protein, it has been shown that soon after secretion it becomes associated with cellular membranes and extracellular matrix [1] [49] [50]. Thus, the pattern of netrin-1 immunoreactivity that we observed suggests that DA neurons express *netrin-1*.

## Discussion

The major finding of this study is that the expression pattern of the netrin-1 receptors DCC and UNC5H by VTA DA neurons shifts dramatically at the peri-pubertal period in both rats and mice. DCC is expressed by TH-positive neurons in the VTA from embryonic life to adulthood. Conversely, UNC5H expression by DA neurons only emerges at the peri-pubertal age and remains elevated throughout adulthood. Western blot analysis confirmed that the relative ratio of DCC to UNC5H (DCC:UNC5H) expression in the VTA shifts significantly toward UNC5H predominance around puberty. The level and pattern of netrin-1 immunoreactivity, however, was very similar between the juvenile and adult period. Together, these results suggest that netrin-1 function may contribute to the plasticity, function and peri-pubertal reorganization of mesocorticolimbic DA systems and that these effects are likely to be controlled by variations in the levels of netrin-1 receptor expression by DA neurons.

The peri-pubertal switch in netrin-1 receptor expression by DA neurons coincides with a) the substantial pubertal increase in density of DA fibers in the mPFC [21] [51] [52]; b) the shift in the relative balance between mesoaccumbens and mesocortical DA function toward greater mPFC DA activity in puberty [53]; and c) the post-pubertal emergence of the DA and behavioral phenotypes displayed by mice that develop with altered levels of netrin-1 receptors [20]. Netrin-1 may therefore participate in the development and function of mesocorticolimbic DA systems by selectively regulating mPFC DA organization.

Further support for the relevance of the pubertal changes in netrin-1 receptor expression in the development of DA systems is provided by the identification of DCC and UNC5H co-expression by single DA neurons. The relative levels of DCC and UNC5H within a single neuron dictate the direction of a neuron's response to netrin-1 [5] [43] [44] [45] [46] [47] [48]. This suggests that the pubertal shift in the DCC:UNC5H ratio represents a developmentally regulated change in the response of DA neurons to netrin-1 that may have a significant impact on the functional organization of DA systems [17] [19] [20].

Spatio-temporal regulation of guidance cue receptor expression is critical for the guidance of neuronal processes, target selection/recognition and synaptogenesis. For instance, during spinal cord development, as commissural axons follow their trajectory toward their final targets, they undergo dynamic changes in expression of netrin-1 and slit receptors at intermediate choice points [54]. A lifespan shift in the expression pattern of netrin-1 receptors similar to that observed in midbrain DA neurons has also been reported in the rat spinal cord [38]. Thus, the organization of CNS networks by netrin-1 during embryonic development and early postnatal life seems to be mediated primarily by DCC. In contrast, from puberty onward, the major netrin-1 transducing receptor appears to be UNC5H. A recent study examining the role of netrin-1 in the formation of hippocampal networks underscores the importance of the regulation of DCC:UNC5H ratios [48]. The axon guidance of mossy fibers to the CA3 region was found to be established initially by DCC-mediated netrin-1 signaling. Subsequently, enhanced neural activity within these fibers led to the recruitment of UNC5A to the cell surface. This shift in DCC:UNC5H ratio triggered mossy fiber sprouting by switching the netrin-1 response to repulsion. Within this context, the peri-pubertal emergence of UNC5H expression by DA neurons that we observed here is likely to be associated with a critical event in the developmental course of this system, most likely related to the organization of mPFC DA circuitry, including modifications in fiber density, shape, and/or distribution.

Indeed, axonal branching and synaptogenesis are crucial determinants of the pattern and strength of neuronal connectivity [55] [56], and these are more pronounced at specific developmental periods. During puberty, the mesocorticolimbic DA system undergoes substantial plasticity and remodeling associated with significant changes in DA neuronal activity [53] [57] [58] [59] [60] [61] [62] [63] [64]. These changes in activity could influence the DCC:UNC5H ratio in DA cells by regulating the levels and cell surface expression of netrin-1 receptors [43] [44] [45] [48] [65]. DCC-mediated netrin-1 signaling has been shown to influence CNS synaptic connectivity [17] [66] [67] and axon branching [67] [68] [69] [70] [71]. Consistent with this, we have found that the onset of UNC5H expression by DA neurons coincides with the emergence of a phenotype in *dcc* +/− mice on sprouting of TH positive terminals in the mPFC; remarkably this effect is not observed prior to puberty (our unpublished observations). It is important to note that arborization of DA axons can be induced by receptor activation at the terminals as well as activation of somatodendritic receptors [72]. Thus, the role of netrin-1 in the organization of mesocorticolimbic DA circuits could indeed be mediated by local events in the VTA. We are currently using *unc-5* homologue mutants to address the potential functional consequences of the peri-pubertal emergence of UNC-5 homologue expression by VTA dopamine neurons at puberty.

Changes in neuronal activity may have different effects on NAcc versus mPFC DA projecting cells. The expression profile of the DA transporter and DA autoreceptors, as well as the extent of VTA DA neuron sensitivity to neurotransmitters all depend on which target the VTA DA cell innervates [73] [74] [75]. Consistent with this, the DA phenotype we observed in *dcc* +/− mice appears to be specific to the mPFC circuit. We found increased expression of TH, but not dopamine β-hydroxylase in the mPFC of adult *dcc* +/− mice, suggesting that there is sprouting of DA, but not noradrenergic, fibers within this region [19]. No differences in TH levels were detected in the NAcc of *dcc* +/− mice. Furthermore, changes in dendritic structure are found in the mPFC, but not the NAcc of *dcc* +/− mice [17]. Significantly, all these phenotypes are only observed after puberty. Although DA neurons projecting to the mPFC and to the NAcc are neuroanatomically and functionally different [75] [76] [77] [78] [79] [80], they cannot be distinguished on the basis of their anatomical distribution within the VTA because they appear to exist as an intermingled population in the PBP, PN and midline nuclei [75] [81]. The sudden and delayed expression of UNC5H by DA neurons, which, in contrast to DCC, appears to be observed within a subset of VTA DA neurons, may be occurring specifically in mPFC projecting cells. This possibility is being currently tested in our laboratory using retrograde tracers.

Finally, changes in DCC and UNC5H expression may directly affect the somatodendritic structure of VTA DA cells and in turn their activity. Recent work from our group shows robust up-regulation of DCC and UNC5 expression in the VTA, but not mPFC or NAcc, following repeated exposure to the stimulant drug amphetamine, using a regimen known to induced structural changes in VTA DA cells [18] [82]. Moreover, blockade of DCC signaling directly into the VTA prevents amphetamine-induced structural and behavioral plasticity [39]. Remarkably, this amphetamine-induced netrin-1/dopamine interaction is reversed when animals are treated before puberty (L. Yetnikoff and C. Flores, unpublished observations).

In conclusion, the present findings demonstrate that netrin-1 receptors are expressed by DA neurons throughout life. While DCC expression predominates until the post-weaning period, UNC5H predominates from puberty onwards. This switch in the relative levels of DCC and UNC5H takes place during a critical developmental period marked by substantial reorganization and vulnerability of the mPFC DA system, which coincides with the onset of symptoms of many psychiatric disorders, including schizophrenia. *dcc* +/− mice exhibit selective alterations in the organization and function of the mesocortical DA circuit and appear to be protected against schizophrenia-like symptoms. Significantly, this phenotype emerges after puberty. Determining the precise role of netrin-1 receptors in the establishment of mesocorticolimbic circuitry may, therefore, help in understanding the mechanisms underlying the development of individual differences in vulnerability to psychopathology.

## Supporting Information

Figure S1 Netrin-1 receptor antibody specificity. A, B) DCC antibody specificity. A) The DCC antibody used in this study detected a single ∼185 kDa band corresponding to DCC in wild-type (+/+) E17 mouse brain, but not in dcc −/− E17 mouse brain. B) Micrographs of coronal midbrain sections from E17 dcc −/− mouse embryos double-labeled with DCC and TH immunoreactivity. No DCC immunoreactivity was observed in dcc −/− embryos, further demonstrating the specificity of the DCC antibody. Note that TH immunopositive neurons are present in the ventral midbrain region of E17 dcc −/− mouse embryos, indicating that DCC is not required for the formation and differentiation of midbrain dopaminergic neurons. Similar results were obtained at earlier embryonic stages (E15, data not shown). The dorsal aspect of coronal sections is on top. Scale bar: 250 µm. C, D) UNC5C antibody specificity. C) UNC5C antiserum specificity. A single band at ∼135 kDa, was detected by Western blot in lysates from both wild-type (+/+) and unc5c −/− cerebella, suggesting that the antiserum can recognize other endogenous UNC-5 homologues in whole cerebellar homogenates in addition to UNC5C. D) Micrographs of coronal sections of cerebella dissected from +/+ and unc5c −/− mice. The pattern of DAB immunolabeling is similar between genotypes, indicating that the UNC5C antiserum also recognizes additional UNC-5 homologues immunohistochemically. Scale bar: 25 µm. E) Schematic representation of the locations of bilateral tissue punches of the VTA for RT-PCR experiments examining the expression of unc-5 homologue mRNAs in this adult mouse somatodendritic DA region (PND60; [41]). unc5c and unc5d homologues are detected in the VTA. The oligonucleotide sequences used are listed in the Materials and Methods section. F) UNC5H immunoreactivity in the VTA of adult wild-type +/+ and unc5c −/− mice. The patterns of immunoreactivity are similar between the two genotypes, indicating that the antiserum also recognizes UNC5D using immunohistochemistry. Animals studied in experiment A: n = 3, B; n = 4, C: n = 5, D: n = 4. Scale bar: 25 µm.
(4.47 MB TIF)

Figure S2 Netrin-1 receptor expression in E17 rat midbrain dopamine neurons. Digitized images of coronal midbrain sections from E17 rat embryos (Similar results were obtained in E15 and E19 rat embryos). Panels on the left hand side show TH expression at different rostro-caudal levels of the E17 rat midbrain. Panels adjacent to the low magnification images of TH immunostaining show co-localization of TH and DCC (A) and absence of co-localization of TH and UNC5H (B) in the ventral midbrain region at the corresponding rostro-caudal levels. In all pictures, the dorsal aspect of coronal sections is on top. Similar results were obtained in the mouse at the corresponding embryonic age (E15, data not shown). Animals studied in experiment: n = 3. Scale bars: 250 µm (images on the extreme left) and 25 µm for other images.
(4.75 MB TIF)

Figure S3 Netrin-1 receptor expression in midbrain dopamine neurons at birth. Digitized images of coronal midbrain hemisections from PND0 rat embryos. In all pictures, dorsal is on top, lateral on the left, and medial on the right. Expression of DCC (A), but not UNC5H (B), was detected in TH immunopositive neurons in the ventral midbrain. Similar results were obtained in PND0 mouse embryos (data not shown). Animals studied in experiment: n = 4. Scale bar: 25 µm.
(8.18 MB TIF)

Figure S4 Netrin-1 receptor expression at post-weaning. Digitized images of coronal midbrain hemisections from PND23 rats at different rostro-caudal levels. In all pictures, dorsal is on top, lateral on the left, and medial on the right. Expression of DCC (A) was detected in TH immunopositive neurons in the VTA throughout the rostro-caudal axis. At this developmental stage, UNC5H expression begins to be detected in some TH positive neurons of the VTA (B). Animals studied in experiment: n = 3. Scale bar: 250 µm.
(6.02 MB TIF)

Figure S5 Netrin-1 receptor expression during the peri-pubertal period. Digitized images of coronal midbrain hemisections from PND35 rats at different rostro-caudal levels. In all pictures, dorsal is on top, lateral on the left, and medial on the right. DCC is expressed in many TH immunopositive neurons in the VTA at all rostro-caudal levels examined (A). At this developmental stage, there is a robust up-regulation of UNC5H expression in both TH negative and TH positive cells in the VTA throughout the rostro-caudal axis (B). Animals studied in experiment: n = 3. Scale bar: 250 µm.
(6.22 MB TIF)

Figure S6 Netrin-1 receptor expression in adulthood. Digitized images of coronal midbrain hemisections from PND90 rats at different rostro-caudal levels. In all pictures, dorsal is on top, lateral on the left, and medial on the right. DCC is expressed in many TH immunopositive neurons in the VTA throughout the rostro-caudal axis (A). At this developmental stage, UNC5H expression is highly expressed in both TH negative and TH positive cells in the VTA at all rostro-caudal levels examined (B). Animals studied in experiment: n = 3. Scale bar: 250 µm.
(6.13 MB TIF)
