# Supplementary figures and images for: Peri-Pubertal Emergence of UNC-5 Homologue Expression by Dopamine Neurons in Rodents
Source: PLoS One. 2010 Jul 8;5(7):e11463. doi: 10.1371/journal.pone.0011463 (PMC2900213; doi:10.1371/journal.pone.0011463)

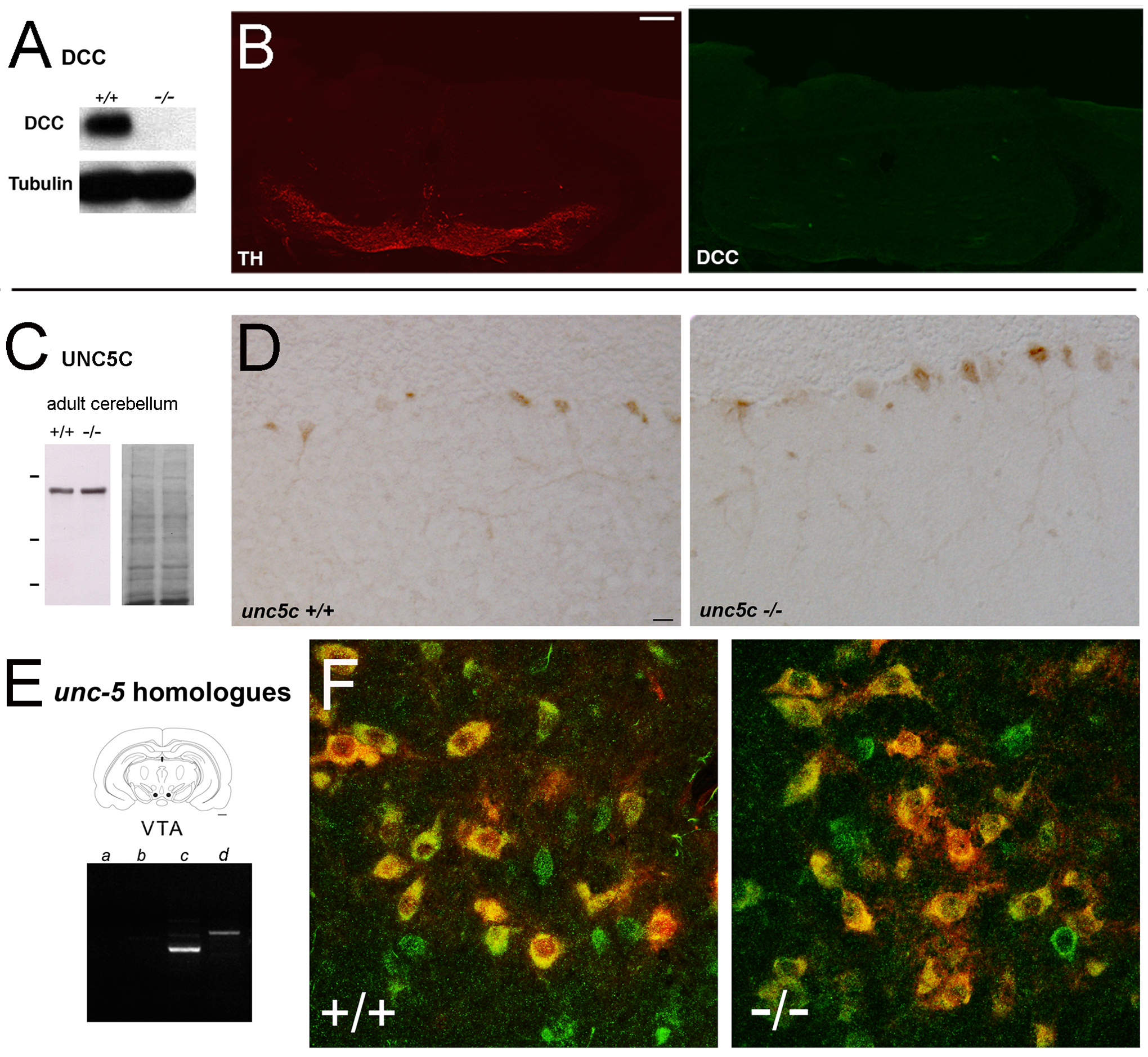

Supplement: Figure S1 — Netrin-1 receptor antibody specificity. A, B) DCC antibody specificity. A) The DCC antibody used in this study detected a single ∼185 kDa band corresponding to DCC in wild-type (+/+) E17 mouse brain, but not in dcc −/− E17 mouse brain. B) Micrographs of coronal midbrain sections from E17 dcc −/− mouse embryos double-labeled with DCC and TH immunoreactivity. No DCC immunoreactivity was observed in dcc −/− embryos, further demonstrating the specificity of the DCC antibody. Note that TH immunopositive neurons are present in the ventral midbrain region of E17 dcc −/− mouse embryos, indicating that DCC is not required for the formation and differentiation of midbrain dopaminergic neurons. Similar results were obtained at earlier embryonic stages (E15, data not shown). The dorsal aspect of coronal sections is on top. Scale bar: 250 µm. C, D) UNC5C antibody specificity. C) UNC5C antiserum specificity. A single band at ∼135 kDa, was detected by Western blot in lysates from both wild-type (+/+) and unc5c −/− cerebella, suggesting that the antiserum can recognize other endogenous UNC-5 homologues in whole cerebellar homogenates in addition to UNC5C. D) Micrographs of coronal sections of cerebella dissected from +/+ and unc5c −/− mice. The pattern of DAB immunolabeling is similar between genotypes, indicating that the UNC5C antiserum also recognizes additional UNC-5 homologues immunohistochemically. Scale bar: 25 µm. E) Schematic representation of the locations of bilateral tissue punches of the VTA for RT-PCR experiments examining the expression of unc-5 homologue mRNAs in this adult mouse somatodendritic DA region (PND60; [41]). unc5c and unc5d homologues are detected in the VTA. The oligonucleotide sequences used are listed in the Materials and Methods section. F) UNC5H immunoreactivity in the VTA of adult wild-type +/+ and unc5c −/− mice. The patterns of immunoreactivity are similar between the two genotypes, indicating that the antiserum also recognizes UNC5D using immuno [file pone.0011463.s001.tif]

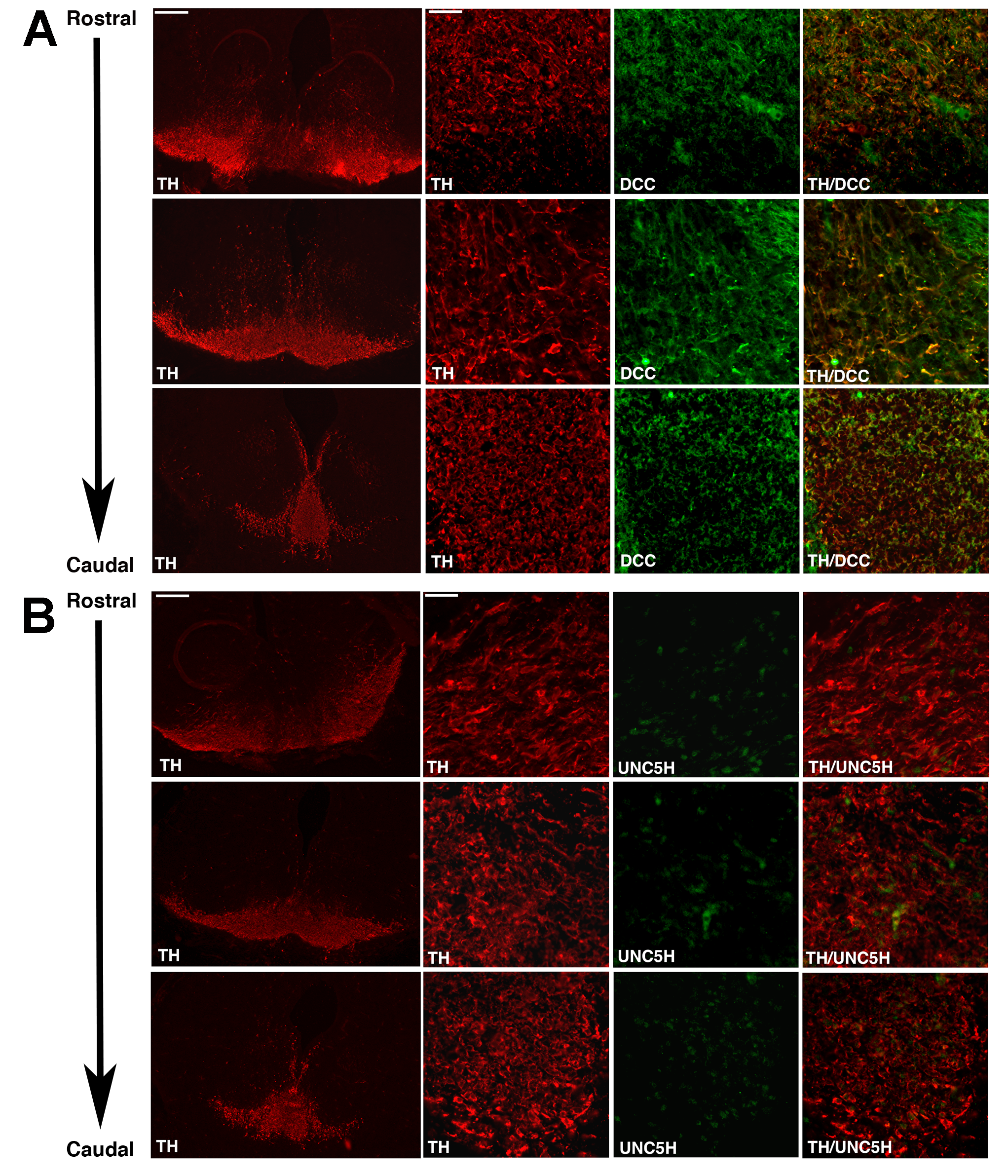

Supplement: Figure S2 — Netrin-1 receptor expression in E17 rat midbrain dopamine neurons. Digitized images of coronal midbrain sections from E17 rat embryos (Similar results were obtained in E15 and E19 rat embryos). Panels on the left hand side show TH expression at different rostro-caudal levels of the E17 rat midbrain. Panels adjacent to the low magnification images of TH immunostaining show co-localization of TH and DCC (A) and absence of co-localization of TH and UNC5H (B) in the ventral midbrain region at the corresponding rostro-caudal levels. In all pictures, the dorsal aspect of coronal sections is on top. Similar results were obtained in the mouse at the corresponding embryonic age (E15, data not shown). Animals studied in experiment: n = 3. Scale bars: 250 µm (images on the extreme left) and 25 µm for other images. (4.75 MB TIF) [file pone.0011463.s002.tif]

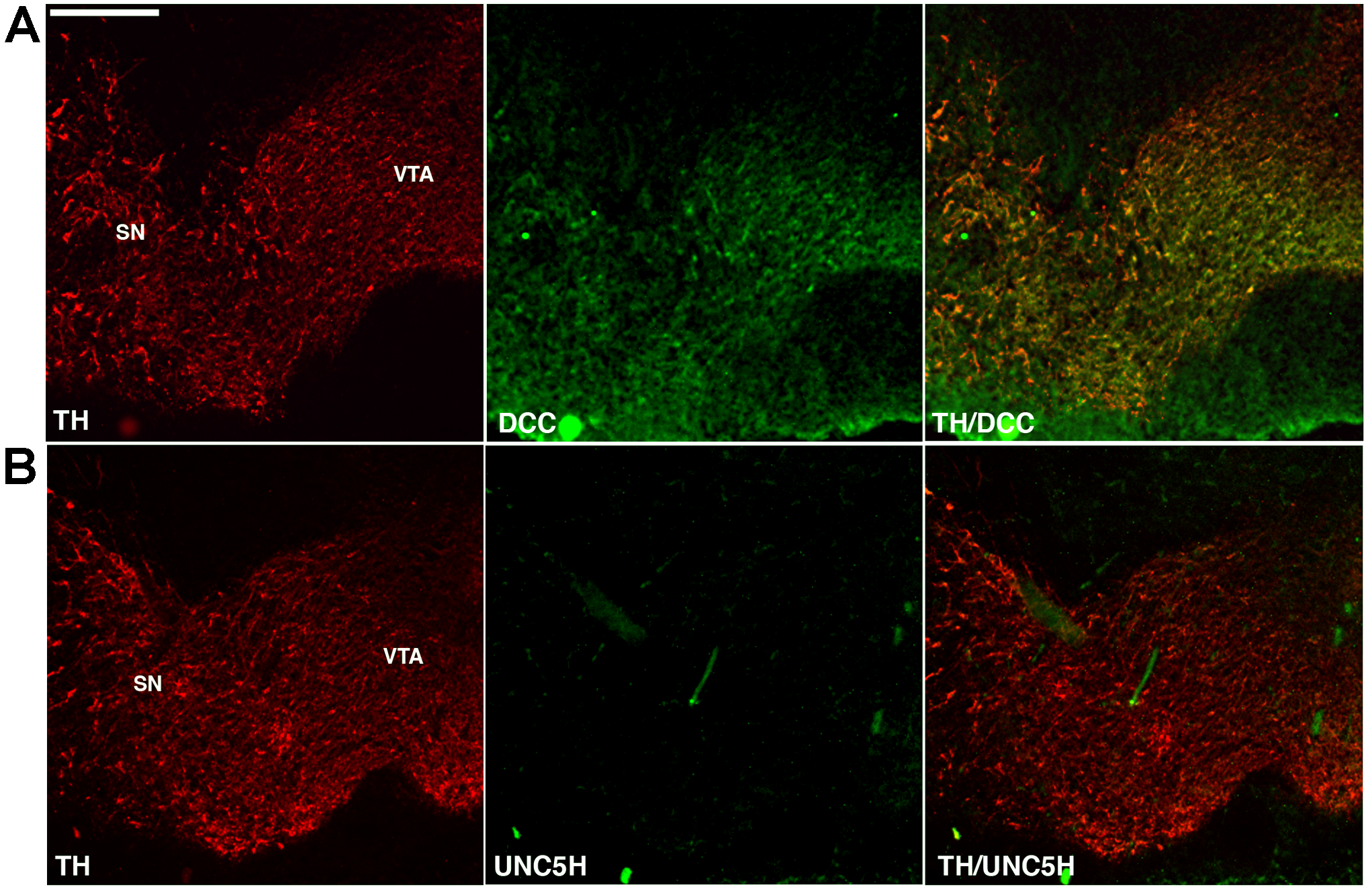

Supplement: Figure S3 — Netrin-1 receptor expression in midbrain dopamine neurons at birth. Digitized images of coronal midbrain hemisections from PND0 rat embryos. In all pictures, dorsal is on top, lateral on the left, and medial on the right. Expression of DCC (A), but not UNC5H (B), was detected in TH immunopositive neurons in the ventral midbrain. Similar results were obtained in PND0 mouse embryos (data not shown). Animals studied in experiment: n = 4. Scale bar: 25 µm. (8.18 MB TIF) [file pone.0011463.s003.tif]

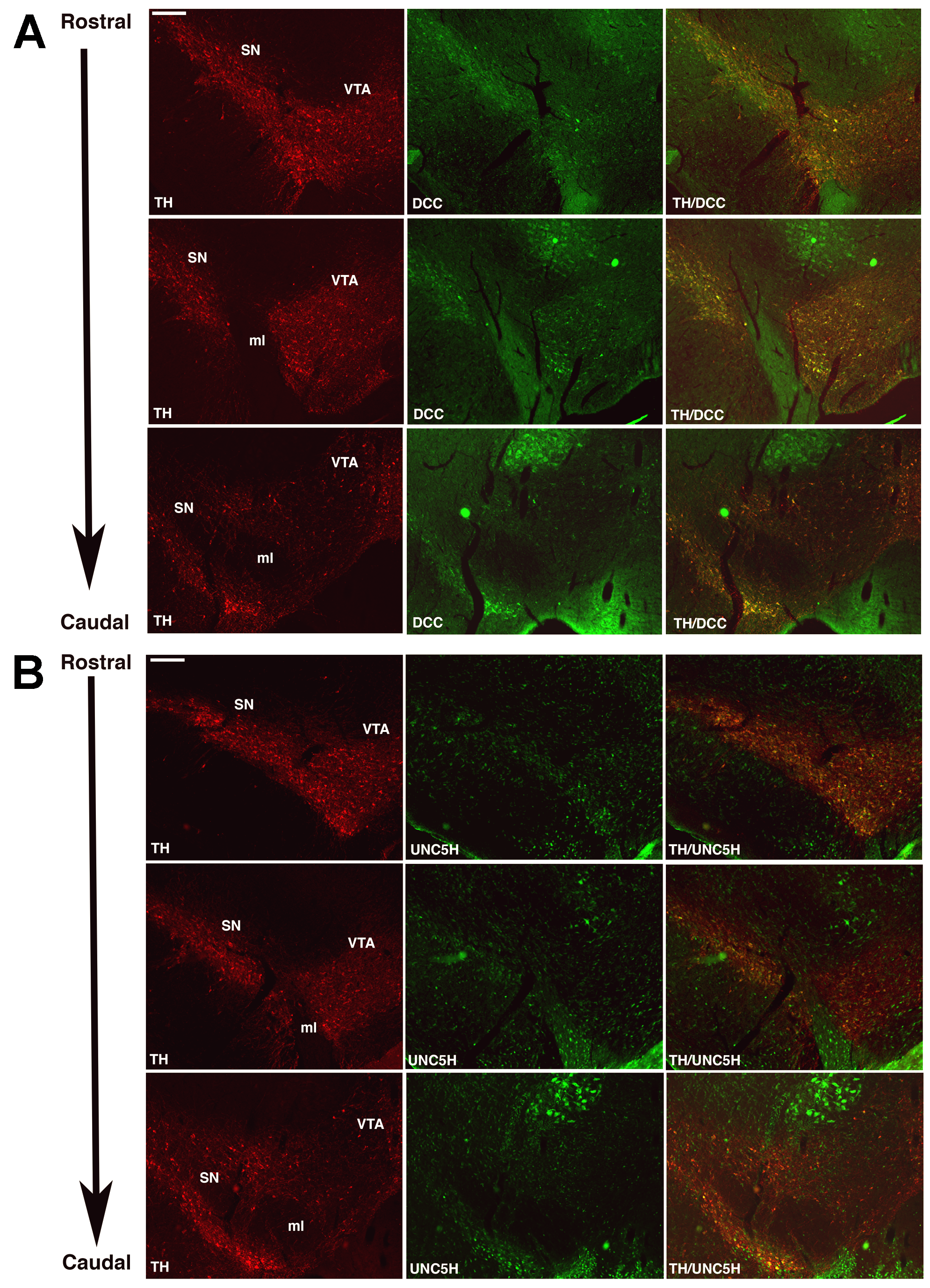

Supplement: Figure S4 — Netrin-1 receptor expression at post-weaning. Digitized images of coronal midbrain hemisections from PND23 rats at different rostro-caudal levels. In all pictures, dorsal is on top, lateral on the left, and medial on the right. Expression of DCC (A) was detected in TH immunopositive neurons in the VTA throughout the rostro-caudal axis. At this developmental stage, UNC5H expression begins to be detected in some TH positive neurons of the VTA (B). Animals studied in experiment: n = 3. Scale bar: 250 µm. (6.02 MB TIF) [file pone.0011463.s004.tif]

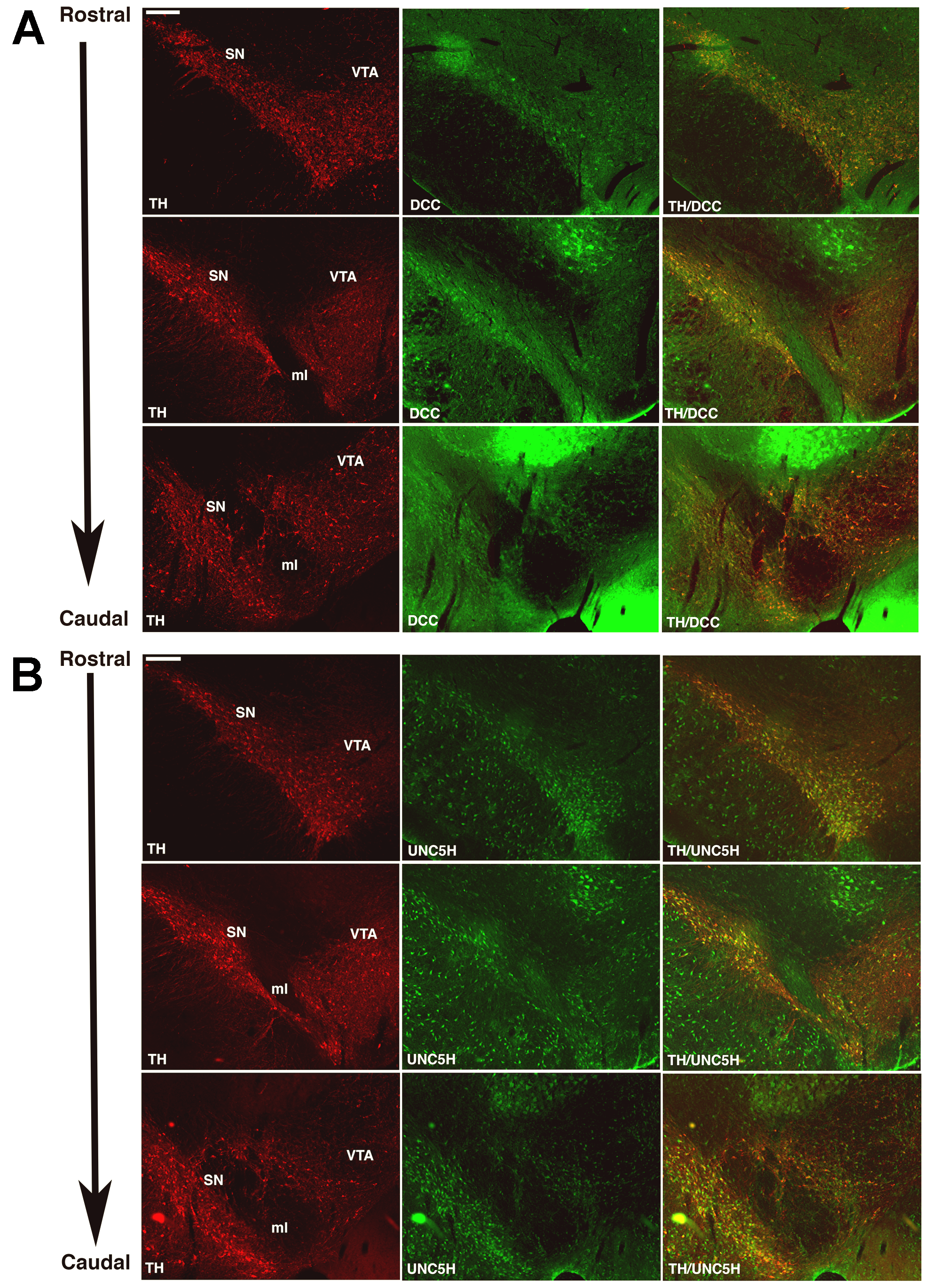

Supplement: Figure S5 — Netrin-1 receptor expression during the peri-pubertal period. Digitized images of coronal midbrain hemisections from PND35 rats at different rostro-caudal levels. In all pictures, dorsal is on top, lateral on the left, and medial on the right. DCC is expressed in many TH immunopositive neurons in the VTA at all rostro-caudal levels examined (A). At this developmental stage, there is a robust up-regulation of UNC5H expression in both TH negative and TH positive cells in the VTA throughout the rostro-caudal axis (B). Animals studied in experiment: n = 3. Scale bar: 250 µm. (6.22 MB TIF) [file pone.0011463.s005.tif]

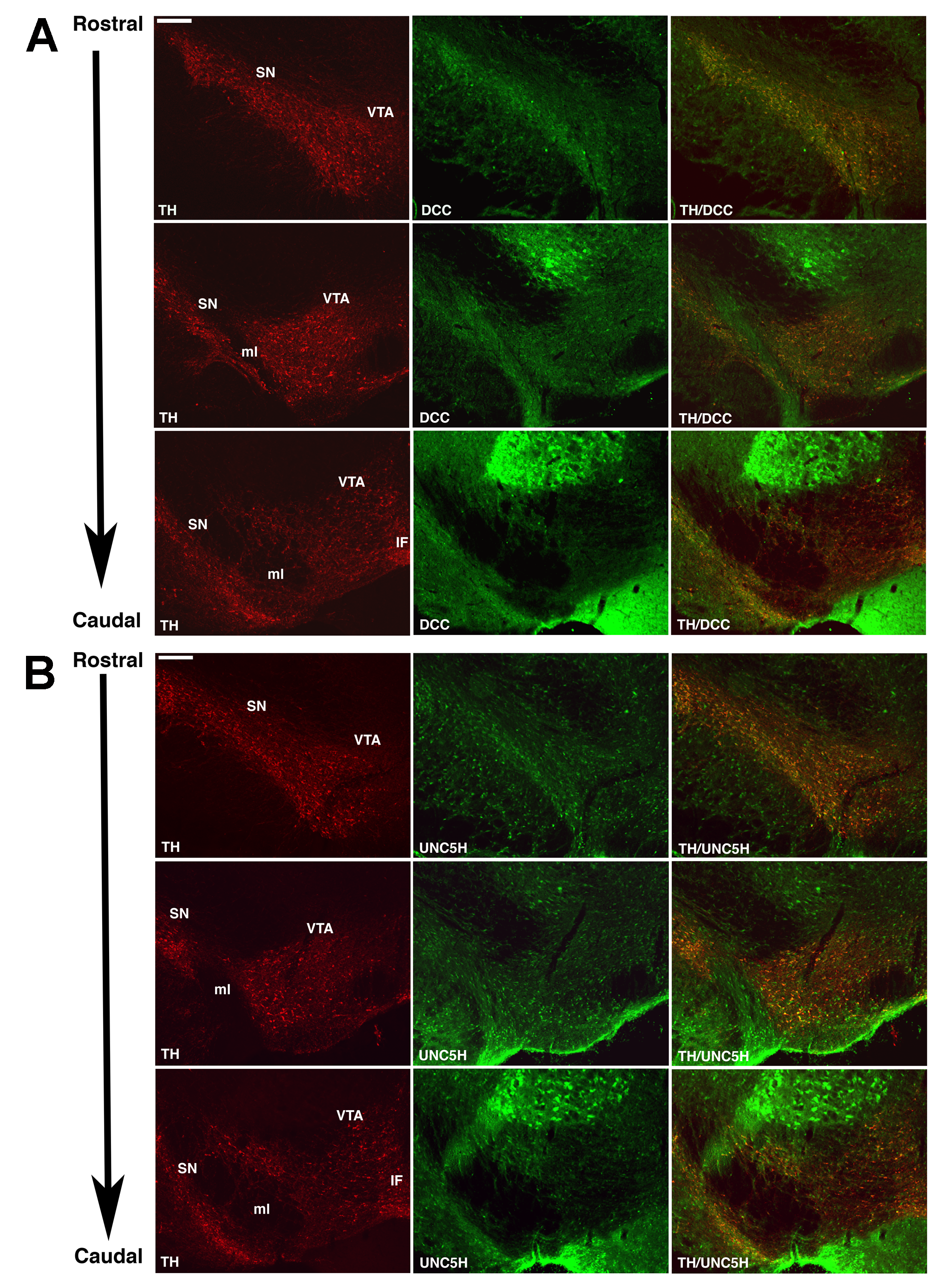

Supplement: Figure S6 — Netrin-1 receptor expression in adulthood. Digitized images of coronal midbrain hemisections from PND90 rats at different rostro-caudal levels. In all pictures, dorsal is on top, lateral on the left, and medial on the right. DCC is expressed in many TH immunopositive neurons in the VTA throughout the rostro-caudal axis (A). At this developmental stage, UNC5H expression is highly expressed in both TH negative and TH positive cells in the VTA at all rostro-caudal levels examined (B). Animals studied in experiment: n = 3. Scale bar: 250 µm. (6.13 MB TIF) [file pone.0011463.s006.tif]
